# Intervenções cirúrgicas e injetáveis na contratura de Dupuytren: protocolo de revisão sistemática

**DOI:** 10.1055/s-0045-1811596

**Published:** 2025-09-08

**Authors:** Rodrigo Guerra Sabongi, Vinicius Ynoe de Moraes, João Baptista Gomes dos Santos, Vinicius Tassoni Civile, Nelson Carvas Junior, Flávio Faloppa

**Affiliations:** 1 Disciplina de Cirurgia da Mão e Membro Superior, Escola Paulista de Medicina, Universidade Federal de São Paulo, São Paulo, SP, Brasil; 2 Programa de Pós-graduação em Saúde Baseada em Evidência, Universidade Federal de São Paulo, São Paulo, SP, Brasil

**Keywords:** colagenases, contratura de Dupuytren, fasciotomia, revisão sistemática, collagenases, Dupuytren contracture, fasciotomy, systematic review

## Abstract

**Objetivo:**

Comparar a eficácia e a segurança de intervenções cirúrgicas e injetáveis para tratamento da contratura de Dupuytren (CD) por meio de revisão sistemática e metodologia de metanálise em rede.

**Métodos:**

Este protocolo segue as diretrizes *Preferred Reporting Items for Systematic Reviews and Meta-Analyses Protocols* (PRISMA-P) e está registrado no banco de dados *The International Prospective Register of Systematic Reviews* (PROSPERO). Devem ser incluídos estudos clínicos randomizados com pacientes adultos com CD e submetidos a intervenções cirúrgicas (ex.: fasciectomia e fasciotomia) ou injetáveis (ex.: colagenase e corticosteroides). Uma busca abrangente deve ser conduzida nas bases de dados MEDLINE, CENTRAL, EMBASE, LILACS, IBECS e registros de estudos clínicos, sem restrições de idioma ou data. Dois revisores devem realizar a triagem, extração de dados e avaliação de risco de viés (RoB2) de forma independente. Os desfechos primários devem incluir melhora funcional e efeitos adversos. Os desfechos secundários devem incluir recidiva, amplitude de movimento e dor. A síntese de dados deve envolver metanálises pareadas e de rede de efeitos aleatórios e a certeza da evidência deve ser avaliada de acordo com a abordagem *Grading of Recommendation, Assessment, Development and Evaluation* (GRADE). As análises de subgrupos devem explorar a heterogeneidade com base em variáveis clínicas e metodológicas.

**Resultados:**

Esta proposta de revisão pretende gerar estimativas comparativas de eficácia e segurança em todas as intervenções elegíveis, incorporando evidências diretas e indiretas. Os desfechos funcionais devem ser sintetizados usando uma hierarquia predefinida de medidas de desfecho relatadas pelo paciente (PROMs, do inglês *patient-reported outcome measures* ) e os tratamentos devem ser classificados com base nos perfis de eficácia e segurança.

**Conclusão:**

Esta revisão sistemática visa preencher lacunas de evidências atuais comparando todas as intervenções relevantes para o tratamento da CD, apoiando a tomada de decisão clínica por meio de síntese robusta de resultados funcionais e de segurança.

## Abstract (en)

**Objective:**

To compare the effectiveness and safety of surgical and injection-based interventions for Dupuytren's disease (DD) using systematic review and network meta-analysis methodology.

**Methods:**

The current protocol follows Preferred Reporting Items for Systematic Reviews and Meta-Analyses Protocols (PRISMA-P) guidelines and is registered in The International Prospective Register of Systematic Reviews (PROSPERO). Randomized controlled trials involving adult patients with DD treated by surgical (e.g., fasciectomy, fasciotomy) or injection-based interventions (e.g., collagenase, corticosteroids) must be included. A comprehensive search must be conducted across MEDLINE, CENTRAL, EMBASE, LILACS, IBECS, and trial registries, without language or date restrictions. Two reviewers must perform independent screening, data extraction, and risk of bias assessment (RoB2). Primary outcomes must include functional improvement and adverse effects. Secondary outcomes must include recurrence, range of motion, and pain. Data synthesis should involve random-effects pairwise and network meta-analyses, with GRADE used to assess certainty of evidence. Subgroup analyses should explore heterogeneity based on clinical and methodological variables.

**Results:**

This proposal of review aims to generate comparative estimates of effectiveness and safety across all eligible interventions, incorporating both direct and indirect evidence. Functional outcomes must be synthesized using a predefined hierarchy of patient-reported outcome measures (PROMs), and treatments were ranked based on efficacy and safety profiles.

**Conclusion:**

This systematic review aims to fill current evidence gaps by comparing all relevant interventions for DD, supporting clinical decision-making through robust synthesis of functional and safety outcomes.

## Introdução

A contratura de Dupuytren (CD) é uma doença fibroproliferativa benigna e progressiva da fáscia palmar caracterizada pela formação de nódulos e cordões que podem evoluir para contraturas em flexão. A CD acomete principalmente as articulações metacarpofalangianas (MCFs) e interfalangianas proximais (IFP). 1 2 A doença é causada pela proliferação e diferenciação anormais de fibroblastos em miofibroblastos contráteis, levando ao espessamento, encurtamento e perda de extensão dos dedos. 1

Embora sua etiologia seja multifatorial, com forte influência genética, vários fatores de risco modificáveis e não modificáveis têm sido associados à doença, incluindo maior idade, sexo masculino, consumo de álcool, tabagismo, diabetes mellitus, epilepsia, doença hepática e ocupações que envolvam trabalho manual ou exposição a vibrações. 3 A prevalência estimada na população geral varia de 4 a 6%, com taxas maiores em indivíduos com ascendência no norte da Europa e em faixas etárias superiores. 2 4 Nos estágios avançados, a doença pode prejudicar significativamente a função das mãos e a qualidade de vida.

As opções para tratamento da CD evoluíram ao longo dos séculos. Intervenções cirúrgicas, como fasciotomia e fasciectomia, são as principais abordagens desde o século XVII. 5 Nos últimos anos, tratamentos menos invasivos têm sido cada vez mais explorados, em especial terapias intralesionais, que visam modificar a progressão da doença em estágios iniciais ou oferecer alternativas à cirurgia. A colagenase injetável de *Clostridium histolyticum* (CCH), que provoca a ruptura enzimática dos cordões de colágeno, demonstrou eficácia em curto prazo na redução de contraturas, principalmente na articulação MCF, e passou a ser bastante utilizada após aprovação regulatória, embora sua disponibilidade varie de acordo com a região. 6 Outros agentes, como corticosteroides injetados em nódulos em estágios iniciais foram associados à regressão parcial ou à estabilização da doença, 7 enquanto terapias biológicas com alvo em vias moleculares, incluindo agentes antifator de necrose tumoral (TNF, do inglês *tumor necrosis factor* ), estão sob investigação e seus primeiros resultados são promissores. 8

Dentre as técnicas cirúrgicas, a fasciotomia percutânea por agulha (PNF) é uma opção minimamente invasiva associada à recuperação rápida e baixa morbidade. No entanto, apresenta alta taxa de recidiva, principalmente em pacientes com doença agressiva. A fasciectomia limitada continua sendo o tratamento padrão para contraturas mais avançadas, oferecendo maior durabilidade, mas ao custo de recuperação mais longa e maiores taxas de complicações. 5 8 Em casos recorrentes ou graves, a dermofasciectomia pode ser indicada, embora envolva o uso de enxerto de pele e apresente maior complexidade cirúrgica e morbidade. A escolha da intervenção depende de múltiplos fatores, incluindo gravidade, acometimento articular, comorbidades, risco de recidiva e experiência do cirurgião. 9

Apesar da existência de diversos tratamentos para a CD, há uma lacuna notável na literatura quanto a comparações abrangentes dessas intervenções. A falta de sínteses comparativas robustas limita a capacidade dos clínicos de identificar quais intervenções oferecem o melhor equilíbrio entre eficácia, segurança e resultados em longo prazo, particularmente no que diz respeito à melhora funcional, efeitos adversos e taxas de recidiva.

As revisões sistemáticas anteriores geralmente se concentraram em modalidades terapêuticas individuaiscomo colagenase, fasciotomia por agulha ou fasciectomia limitada-excluindo outras técnicas cirúrgicas e injetáveis relevantes, como corticosteroides ou agentes biológicos experimentais. 10 11 12 13 14 Uma única revisão conduziu uma metanálise em rede e, mesmo assim, a comparação foi restrita a três intervenções. 15 Apenas uma revisão Cochrane, publicada em 2015, tentou comparações mais amplas, mas incluiu somente técnicas cirúrgicas e não incorporou modalidades mais recentes. 16

Diversas revisões recentes também apresentaram limitações metodológicas importantes que dificultam sua aplicabilidade na tomada de decisões clínicas. Muitas restringiram a inclusão a estudos publicados em inglês ou obtidos apenas de bases de dados tradicionais, talvez ignorando dados relevantes de outras regiões ou idiomas. 10 11 15 Além disso, a seleção e de medidas de desfechos relatados pelo paciente (PROMs, do inglês *patient-reported outcome measures* ) têm sido inconsistentes, muitas vezes sem hierarquia ou justificativa clara. 11 12 13 Várias revisões recentes não incluíram metanálise em rede, restringindo sua capacidade de comparar intervenções além de análises pareadas diretas. 10 11 12 13 14

Por fim, a maioria das revisões existentes estão agora metodologicamente desatualizadas, tendo concluído as suas pesquisas bibliográficas há mais de 5 anos, antes da publicação de vários estudos clínicos randomizados importantes.

Coletivamente, essas limitações reforçam a necessidade de uma revisão sistemática atualizada, abrangente e com metodologia rigorosa para subsidiar a tomada de decisões clínicas baseadas em evidências. Este protocolo descreve a metodologia para uma revisão sistemática e metanálise em rede com o objetivo de comparar a eficácia e a segurança de intervenções cirúrgicas e injetáveis para o tratamento da CD.

## Materiais e Métodos

Esta revisão foi conduzida de acordo com as diretrizes *Preferred Reporting Items for Systematic Reviews and Meta-Analyses for Network Meta-Analyses* (PRISMA-NMA). 17 Este estudo foi registrado no banco de dados *The International Prospective Register of Systematic Reviews* (PROSPERO), sob o número CRD42023439436. O desenvolvimento deste protocolo utilizou a lista de verificação *Preferred Reporting Items for Systematic Reviews and Meta-Analyses Protocol* (PRISMA-P). 18 Quaisquer alterações substanciais serão documentadas e atualizadas no registro PROSPERO.

### Critérios para a Consideração de Estudos

#### População do Estudo e Delineamento Experimental

A revisão proposta deve incluir estudos clínicos randomizados (ECRs) que avaliaram intervenções cirúrgicas e injetáveis para tratamento da CD. Os estudos elegíveis devem incluir participantes com 18 anos ou mais, diagnosticados com CD e submetidos a intervenções cirúrgicas ou injetáveis. Para garantir a avaliação dos resultados em longo prazo, apenas estudos com período de acompanhamento superior a 3 meses devem ser incluídos. Não deve haver restrições quanto ao *status* da publicação ou idioma, permitindo a inclusão de estudos publicados ou não.

#### Definições e Medidas de Desfecho

Os estudos devem ter um braço de comparação com infiltração e/ou tratamento cirúrgico. Estudos que compararam as seguintes intervenções serão elegíveis para inclusão nesta revisão:

- Intervenções cirúrgicas: fasciectomia total ou parcial, fasciotomia percutânea, entre outras.
- Intervenções de infiltração: quaisquer tratamentos que utilizem substâncias ativas como, CCH ou corticosteroides, entre outros.

Os desfechos primários da revisão proposta se concentrarão na melhora funcional e nos efeitos adversos. A melhora funcional pode ser avaliada por meio de escalas relatadas pelo paciente, seguindo uma hierarquia predefinida de instrumentos, de mais a menos específicas para a doença ( Tabela 1 ). 19 20 21 22 23 24

**Tabela 1:** Hierarquia das medidas de PROMs para avaliação de melhora funcional na CD

Classificação	Instrumento	
1	SDSS 19	
2	DIF-CHUM 20	
3	URAM 21	
4	Questionário DASH e/ou QuickDASH 22	
5	MHQ e/ou briefMHQ 23	
6	CHFS 24	

**Abreviações:** CD, contratura de Dupuytren; CHFS, Cochin Rheumatoid Arthritis Hand Function Scale; DASH, Disability of the Arm, Shoulder, and Hand Questionnaire; DIF-CHUM, Dupuytren's Contracture Impact on Function - Centre Hospitalier de l'Université de Montréal; MHQ, Michigan Hand Questionnaire; PROM, desfecho relatadas pelo paciente; SDSS, Southampton Dupuytren's Scoring Scheme; URAM, Unité Rhumatologique des Affections de la Main.

Os efeitos adversos devem ser categorizados em complicações menores e maiores e classificados por ordem de interesse para análise. Complicações menores incluem infecção superficial, deiscência da ferida, retardo na cicatrização e hematoma. Complicações maiores consistem em infecção profunda, necessidade de nova cirurgia, cobertura cutânea, isquemia, necrose, lesão nervosa, ruptura de tendão, internação em unidade de terapia intensiva e óbito. As seguintes complicações serão priorizadas na análise, em ordem decrescente de interesse: lesão nervosa, retardo na cicatrização, necessidade de reoperação, infecção superficial e infecção profunda.

Os desfechos secundários incluirão a amplitude de movimento, medida pelo ângulo da contratura, amplitude total de movimento ou estágio de Tubiana. A dor será avaliada pela escala visual analógica (EVA). A recidiva será definida como mais de 20 graus de contratura em qualquer articulação tratada 1 ano após a intervenção em comparação a 6 semanas após o tratamento; cada articulação tratada será avaliada individualmente. 25

#### Métodos de Pesquisa para Identificação de Estudos

Os estudos relevantes devem identificados por meio de buscas nas bases de dados eletrônicas e registros de estudos clínicos: Cochrane Library, Medline, Embase, Lilacs, Ibecs, ClinicalTrials.gov e *International Clinical Trials Registry Platform* da Organização Mundial da Saúde. Essas buscas serão realizadas sem restrições de idioma ou data de publicação. O Anexo 1 mostra a estratégia completa de busca para cada banco de dados. Além disso, as buscas manuais devem incluir a revisão de listas de referências de resumos e anais de conferências relevantes e especialistas e autores serão contatados para identificar estudos não publicados ou em andamento.

### Coleta e Análise de Dados

#### Seleção de Estudos

O processo de triagem deve ser conduzido utilizando o software Rayyan (Qatar Foundation). Dois autores devem selecionar, de forma independente, títulos e resumos para identificar estudos com potencial elegibilidade com base em critérios predefinidos. Os artigos completos dos estudos incluídos na triagem inicial devem ser recuperados, seguindo avaliação de sua elegibilidade para inclusão final. Em caso de discordância, um terceiro revisor deve ser consultado para resolver conflitos. O processo de seleção deve ser documentado em um fluxograma PRISMA, detalhando os motivos para a exclusão de estudos.

#### Análise e Síntese de Dados

A extração e o gerenciamento de dados devem ser conduzidos de forma independente por dois autores usando um formulário padronizado no Microsoft Excel (Microsoft Corp.). Os dados extraídos incluem métodos de estudo (delineamento experimental, definição), dados de identificação (financiamento, país, detalhes do autor), características dos participantes (número de indivíduos randomizados, analisados e perdidos ao acompanhamento), dados basais (idade, sexo, gravidade da doença, índice de massa corporal, duração da doença), intervenções (descrição, tamanhos de grupo), medidas de desfecho (tipo, escala, unidade), características do delineamento experimental e avaliação do risco de viés. Tabelas com resumos das características do estudo e do risco de viés devem ser criadas. Para desfechos contínuos, médias, desvios padrão (DPs) e números de participantes serão extraídos, com transformação de dados conforme necessário. Para desfechos dicotômicos, taxas de eventos e números de participantes serão extraídos. A análise de intenção de tratar será aplicada sempre que possível. Discrepâncias devem ser resolvidas por consenso e os dados finais exportados para RStudio (Posit Software, PBC) para análise.

Para desfechos contínuos, médias, DPs e números de participantes devem ser extraídos. Caso sejam relatadas medidas alternativas de dispersão (ex.: intervalos de confiança ou erros-padrão), os DPs devem ser calculados de acordo com o Manual Cochrane. Diferenças médias padronizadas (SMDs) devem ser utilizadas para desfechos relatados em diferentes escalas, com resultados expressos como intervalos de confiança (ICs) de 95%. Para desfechos dicotômicos, as taxas de eventos e o número de participantes devem ser extraídos para calcular razões de chances (ORs) com ICs de 95%.

A unidade de análise considerada é cada participante. Para estudos que relatam múltiplos pontos temporais, os dados do acompanhamento mais longo devem ser extraídos. Para dados ausentes, os autores do estudo devem ser contatados. Se não estiverem disponíveis, deve-se prosseguir com os dados existentes e as análises de sensibilidade avaliarão o possível impacto. A análise por intenção de tratar (ITT) deve ser utilizada sempre que possível.

A heterogeneidade deve ser avaliada com base nas características dos participantes, intervenções e desfechos. Os resultados estatísticos devem ser avaliados por meio de gráficos de floresta ( *forest plots* ) e quantificada pela estatística I ^2^ , com um teste do qui-quadrado ( *p* < 0,1) para detecção de significância. A heterogeneidade deve ser classificada como não importante (0–40%), moderada (30–60%), substancial (50–74%) ou considerável (75–100%). Análises de subgrupos podem ser conduzidas para explorar possíveis fontes de heterogeneidade.

Tabelas com resumo dos resultados devem ser preparadas para todos os desfechos, utilizando o comparador de referência mais apropriado. A certeza da evidência deve ser avaliada utilizando a abordagem Grading of Recommendations, Assessment, Development and Evaluation (GRADE) por dois autores. A certeza deve ser rebaixada com base em fatores como limitações do estudo, inconsistência e viés de publicação, com os resultados categorizados como alto, moderado, baixo ou muito baixo. As discordâncias devem ser resolvidas por consenso, com descrição das justificativas para os rebaixamentos em notas de rodapé.

As análises de subgrupos devem investigar a heterogeneidade por idade, comorbidades (ex.: diabetes, epilepsia, alcoolismo, tabagismo), duração da doença, gravidade (estágio de Tubiana/amplitude total de movimento) e técnicas cirúrgicas (ex., palma aberta, McCash, incisão de Bruner, enxerto de pele, zetaplastia). Análises de sensibilidade devem ser conduzidas para testar a robustez dos resultados, com foco na inclusão de estudos não publicados e com alto risco de viés.

### Avaliação de Qualidade

O risco de viés de cada estudo incluído deve ser avaliado de forma independente por dois autores, usando a versão 2 da ferramenta Cochrane *Risk of Bias* (RoB2, Cochrane Collaboration), seguindo as recomendações do *Cochrane Handbook for Systematic Reviews of Interventions* , versão 6.3. Quaisquer discordâncias devem ser resolvidas por consenso. A avaliação deve se concentrar no viés em vários domínios: viés decorrente do processo de randomização, desvios das intervenções pretendidas, ausência de dados de desfechos, medida dos desfechos e seleção dos resultados relatados. Para cada domínio, as respostas às perguntas de sinalização resultarão em um julgamento de “baixo risco de viés”, “algumas preocupações” ou “alto risco de viés”. Uma avaliação geral deve ser fornecida com base no algoritmo da ferramenta RoB2.

O viés de publicação deve ser avaliado usando gráficos de funil e teste de Egger desde que haja pelo menos 10 estudos disponíveis para comparação direta. Metanálises em rede devem ser conduzidas para comparações diretas e indiretas de intervenções. Metanálises em rede frequencistas de efeitos aleatórios e metanálises pareadas devem avaliar a transitividade e informarão a força da evidência. A coerência entre evidências diretas e indiretas deve ser avaliada usando o método de nó dividido.

As classificações de tratamento devem ser calculadas com base no escore P, um análogo à superfície sob a curva de classificação cumulativa (SUCRA), para determinar a probabilidade de cada intervenção ser a mais eficaz. Os valores dos escores podem ser usados para classificar os tratamentos de acordo com estimativas pontuais ponderadas por precisão. Todas as análises devem ser realizadas usando o pacote netmeta, versão 2.8-0, em R (R Foundation for Statistical Computing).

### Ética e Disseminação

A revisão sistemática proposta segue as diretrizes do Conselho Nacional de Saúde do Brasil. Como envolve a síntese de dados previamente publicados e anonimizados, a revisão não requer revisão formal ou aprovação pelo Comitê de Ética em Pesquisa. Uma Declaração de Responsabilidade confirmando esta isenção está disponível mediante solicitação. Os resultados desta revisão serão disseminados por meio de publicação em periódicos revistos por pares e apresentações em congressos científicos. Os dados serão disponibilizados mediante solicitação razoável, seguindo as melhores práticas de curadoria e depósito.

## Discussão

Como uma doença fibroproliferativa progressiva, a CD afeta significativamente a funcionalidade das mãos e a qualidade de vida. 5 26 Apesar da disponibilidade de múltiplos tratamentos, como fasciotomia percutânea por agulha, fasciectomia e injeções de CCH, os resultados ainda são inconsistentes e as taxas de recidiva são altas. 6 27 A ausência de definições padronizadas de recidiva e a heterogeneidade nas medidas de desfechos complicam as comparações diretas entre os estudos existentes. 28 29 30 Este protocolo visa abordar essas lacunas ao empregar uma abordagem sistemática para síntese de evidências comparativas robustas.

O presente estudo adota a metodologia PRISMA para assegurar transparência e reprodutibilidade. 17 Os principais pontos fortes são a aplicação da metanálise em rede, que permite comparações diretas e indiretas entre múltiplas intervenções, e a integração de análises planejadas de subgrupos – considerando variáveis como idade do paciente, comorbidades e gravidade da doença – para melhorar a aplicabilidade clínica. No entanto, espera-se que a revisão enfrente desafios inerentes à literatura, particularmente a heterogeneidade nos relatos de desfechos e a variabilidade na qualidade dos estudos. 13 28 30 Essa variabilidade representa uma possível fonte de viés que pode afetar a robustez das análises planejadas. Esses fatores podem introduzir viés e influenciar a força das evidências resultantes.

A necessidade de uma nova revisão sistemática sobre este tema é reforçada pelas limitações metodológicas observadas em estudos recentes. A maioria dos estudos disponíveis avaliou apenas duas ou três intervenções, tipicamente PNF, colagenase e fasciectomia limitada, excluindo assim opções de tratamento relevantes. Além disso, os relatos de desfechos têm sido heterogêneos, com pouco ou nenhum uso de hierarquias estruturadas de PROM. A maioria das revisões também realizou suas buscas bibliográficas há mais de 5 anos e, assim, não discutiu diversos estudos clínicos randomizados de alta qualidade publicados mais recentemente. 13 15 16 Coletivamente, essas limitações destacam uma lacuna na literatura que justifica a implementação de uma revisão sistemática abrangente, atualizada e de metodologia robusta, como proposto neste protocolo.

Os achados da revisão proposta podem embasar decisões terapêuticas e aprimorar o manejo clínico da CD. Ao abordar lacunas críticas, em especial a ausência de definições padronizadas e medidas de desfecho consistentes de melhora funcional e efeitos adversos, este estudo pode contribuir para avaliações mais uniformes e confiáveis em pesquisas futuras.

**Anexo 1:** 

Estratégia de busca para CENTRAL (via Cochrane Library) #1. Descritor MeSH: [ *Dupuytren Contracture* (contratura de Dupuytren)] explodir todas as árvores #2. ( *Contracture, Dupuytren* [contratura de Dupuytren]) *OR* (OU) ( *Dupuytren's Contracture* [contratura de Dupuytren]) *OR* (OU) ( *Contracture, Dupuytren's* [contratura de Dupuytren]) *OR* (OU) ( *Dupuytrens Contracture* [contratura de Dupuytren]) *OR* (OU) (Fibromatosis, Palmar) *OR* (OU) (Palmar Fibromatosis) *OR* (OU) ( *Dupuytren's Disease* [contratura de Dupuytren]) *OR* (OU) ( *Dupuytren Disease* [contratura de Dupuytren]) *OR* (OU) ( *Dupuytrens Disease* [contratura de Dupuytren]) #3. #1 *AND* (E) #2 Estratégia de busca para Medline (via PubMed) #1. “ *Dupuytren Contracture* [contratura de Dupuytren]”[Mesh] *OR* (OU) ( *Contracture, Dupuytren* [contratura de Dupuytren]) *OR* (OU) ( *Dupuytren's Contracture* [contratura de Dupuytren]) *OR* (OU) (Contracture, Dupuytren's) *OR* (OU) ( *Dupuytrens Contracture* [contratura de Dupuytren]) *OR* (OU) ( *Fibromatosis, Palmar* [Fibromatose Palmar]) *OR* (OU) ( *Palmar Fibromatosis* [Fibromatose Palmar]) *OR* (OU) ( *Dupuytren's Disease* [contratura de Dupuytren]) *OR* (OU) ( *Dupuytren Disease* [contratura de Dupuytren]) *OR* (OU) ( *Dupuytrens Disease* [contratura de Dupuytren]) #2. (“ *clinical* (clínico)” [ *Title/Abstract* (Título/Resumo)] *AND* (E) “ *trial* (estudo)” [ *Title/Abstract* (Título/Resumo)]) *OR* (OU) “ *clinical trials as topic* (estudos clínicos como tópico” [ *MeSH Terms* (Termos MeSH)] *OR* (OU) “ *clinical trial* (estudo clínico)” [ *Publication Type* (Tipo de Publicação)] *OR* (OU) “ *random* * (aleatório*)”[ *Title/Abstract* (Título/Resumo)] *OR* (OU) “ *random allocation* ( *alocação aleatória* )”[ *MeSH Terms* (Termos MeSH)] *OR* (OU) “ *therapeutic use* (uso terapêutico)”[ *MeSH Subheading* (Subtítulo MeSH)] #3. #1 *AND* (E) #2 Estratégia de busca para Embase (via Elsevier) #1. *'Dupuytren contracture* (contratura de Dupuytren) *'* / *exp* *OR* (OU) *'contracture, dupuytren* (contratura de Dupuytren)' *OR* (OU) *'contracture, palmar fascia* (contratura da fáscia palmar)' *OR* (OU) *'dupuytren disease* (contratura de Dupuytren)' *OR* (OU) *'Dupuytren's contracture* (contratura de Dupuytren) *'* *OR* (OU) *'palmar fascial contracture* (contratura da fáscia palmar) *'* *OR* (OU) *'palmar fibromatosis* (fibromatose palmar)' *OR* (OU) *'Dupuytren contracture* (contratura de Dupuytren)' #2. *random* * (aleatório*) *OR* (OU) *factorial* * (fatorial*) *OR* (OU) *crossover* * (transversal) *OR* (OU) ( *cross over* * [transversal*]) *OR* (OU) placebo* *OR* (OU) 'placebo'/exp *OR* (OU) ( doubl* blind* [duplo-cego*]) *OR* (OU) ( singl* blind* [cego simples*) *OR* (OU) ( assign* allocate* [atribuição* alocação*) *OR* (OU) *volunteer* * (voluntário) *OR* (OU) ' *volunteer* (voluntário) '/exp *OR* (OU) ( *crossover procedure* [procedimento transversal]) *OR* (OU) ( *double 'blind'* [duplo-cego]/exp) *procedure* (procedimento) *OR* (OU) ( *randomized controlled trial* [estudo controlado randomizado) *OR* (OU) ( *single 'blind'* [cego simples]/exp) *OR* (OU) *procedure* (procedimento) *OR* (OU) ( *controlled clinical trial* [ensaio clínico controlado]) *OR* (OU) ( *clinical trial* [estudo clínico]) #3. #1 *AND* (E) #2 #4. #3 *AND* (E) [embase]/lim *NOT* (NÃO) ([embase]/lim *AND* (E) [medline]/lim) Estratégia de busca para BVS Regional Portal #1. MH:”Contratura de Dupuytren” *OR* (OU) (Contratura de Dupuytren) *OR* (OU) (Dupuytren Contracture) *OR* (OU) (Contractura de Dupuytren) *OR* (OU) (Doença de Dupuytren) *OR* (OU) (Fibromatose Palmar) *OR* (OU) MH:C04.557.450.565.590.340.173$ *OR* (OU) MH:C05.651.197.270$ *OR* (OU) MH:C17.300.270$ #2. ((AB:ENSAIO$) *OR* (OU) (TW:ENSAIO$) *OR* (OU) (TI:ENSAIO$) *OR* (OU) (TRIAL$) *OR* (OU) (Ensayo$) *OR* (OU) (TW:Ensaio$ Clínico$ Controlado$) *OR* (OU) (AB:Ensaio$ Clínico$ Controlado$) *OR* (OU) (TI:Ensaio$ Clínico$ Controlado$) *OR* (OU) (ENSAIO$ CLINICO$) *OR* (OU) (Ensayo$ Clínico$) *OR* (OU) (Clinical Trial$) *OR* (OU) (ECR) *OR* (OU) (RCT) *OR* (OU) (ESTUDO$ RANDOMIZADO$ CONTROLADO$) *OR* (OU) (RANDOMISED CONTROLLED TRIAL$) *OR* (OU) (RANDOMIZADO$) *OR* (OU) (randomized controlled TRIAL) *OR* (OU) (QUASE RANDOMIZADO$) *OR* (OU) (ENSAIO$ QUASE RANDOMIZADO$) *OR* (OU) (ENSAIO$ CLINICO$ ALEATORIO$) *OR* (OU) (ENSAIO$ CLINICO$ CONTROLADO$) *OR* (OU) (RANDOMIZED SINGLE-BLIND CLINICAL TRIAL$) *OR* (OU) (RANDOMIZED SINGLE BLIND CLINICAL TRIAL$) *OR* (OU) (ensaio$ clínico$ randomizado$ simples cego) *OR* (OU) (CONTROL$) *OR* (OU) (RANDOM$) *OR* (OU) (PLACEBO$) *OR* (OU) (CUSTO) *OR* (OU) (EFICA$) *OR* (OU) (open controlled study) *OR* (OU) (TI:(Estudio doble ciego)) *OR* (OU) (ti:(Double-blind study)) *OR* (OU) (PT:”RANDOMIZED CONTROLLED TRIAL”) *OR* (OU) (“ENSAIO CLINICO CONTROLADO ALEATÓRIO”) *OR* (OU) (“ENSAYO CLINICO CONTROLADO ALEATORIO”) *OR* (OU) (“RANDOMIZED CONTROLLED CLINICAL TRIAL”) *OR* (OU) (“ENSAIO CONTROLADO ALEATÓRIO”) *OR* (OU) (“ENSAIO CLINICO CONTROLADO RANDOMIZADO”) *OR* (OU) (“ENSAYO CONTROLADO ALEATORIO”) *OR* (OU) (“ENSAYO CLINICO CONTROLADO RANDOMIZADO”) *OR* (OU) MH:V03.200.700$ *OR* (OU) PT:”CONTROLLED CLINICAL TRIAL” *OR* (OU) “ENSAIO CLINICO CONTROLADO” *OR* (OU) “ENSAYO CLINICO CONTROLADO” *OR* (OU) MH:V03.200.500$ *OR* (OU) MH:”RANDOMIZED CONTROLLED TRIALS AS TOPIC” *OR* (OU) “ENSAIOS CLINICOS CONTROLADOS ALAEATORIOS COMO ASSUNTO” *OR* (OU) “ENSAYOS CLINICOS CONTROLADOS ALEATORIOS COMO ASSUNTO” *OR* (OU) “CLINICAL TRIALS, RANDOMIZED” *OR* (OU) “ENSAIOS CLINICOS TERAPEUTICOS COMO ASSUNTO” *OR* (OU) “ENSAYOS CLINICOS CONTROLADOS COMO TEMA” *OR* (OU) MH:E05.318.760.535.365.500$ *OR* (OU) MH:E05.337.250.365.500$ *OR* (OU) MH:N05.715.360.775.235.387.500$ *OR* (OU) MH:N06.850.520.450.535.365.500$ *OR* (OU) MH:SP5.001.012.038.059.055$ *OR* (OU) MH:”RANDOM ALLOCATION” *OR* (OU) “DISTRIBUIÇÃO ALEATÓRIA” *OR* (OU) “DISTRIBUICION ALEATORIA” *OR* (OU) RANDOMIZATION *OR* (OU) “DISTRIBUIÇÃO CASUAL” *OR* (OU) RANDOMIZAÇÃO *OR* (OU) “ASIGNACION AL AZAR” *OR* (OU) RANDOMIZACION *OR* (OU) MH:E05.318.780.700$ *OR* (OU) MH:E05.581.500.805$ *OR* (OU) MH:N05.715.360.780.675$ *OR* (OU) MH:N06.850.520.445.700$ *OR* (OU) MH:”DOUBLE- BLIND METHOD” *OR* (OU) “DOUBLE-MASKED STUDY” *OR* (OU) “METODO DUPLO- CEGO” *OR* (OU) “METODO DOBLE CIEGO” *OR* (OU) “ESTUDIO DOBLE ENMASCARADO” *OR* (OU) MH:E05.318.780.300$ *OR* (OU) MH:E05.581.500.300$ *OR* (OU) MH:N05.715.360.780.320$ *OR* (OU) MH:N06.850.520.445.300$ *OR* (OU) MH:SP5.001.012.038.059.065$ *OR* (OU) MH:”SINGLE-BLIND METHOD” *OR* (OU) “METODO SIMPLES-CEGO” *OR* (OU) “METODO SIMPLE-CIEGO” *OR* (OU) “SINGLE- MASKED STUDY” *OR* (OU) “ESTUDIO SIMPLE ENMASCARADO” *OR* (OU) “METODO SIMPLECIEGO” *OR* (OU) MH:E05.318.780.850$ *OR* (OU) MH:N05.715.360.780.730$ *OR* (OU) MH:N06.850.520.445.850$ *OR* (OU) MH:SP5.001.012.038.059.070$ *OR* (OU) PT:”CLINICAL TRIAL” *OR* (OU) “ENSAIO CLINICO” *OR* (OU) “ENSAYO CLINICO” *OR* (OU) MH:V03.200$ *OR* (OU) MH:PLACEBOS *OR* (OU) PLACEBO$ *OR* (OU) MH:D26.660$ MH:E02.785$ or MH:HP3.073.433$ or MH:HP7.007.130$ *OR* (OU) MH:”RESEARCH DESIGN” *OR* (OU) “PROJETOS DE PESQUISA” *OR* (OU) “EXPERIMENTAL DESIGN” *OR* (OU) “PROYECTOS DE INVESTIGACION” *OR* (OU) “DESENHO EXPERIMENTAL” *OR* (OU) “PROYECTO EXPERIMENTAL” *OR* (OU) MH:E05.581.500$ *OR* (OU) MH:H01.770.644.728$ *OR* (OU) MH:SH1.020.010.070$ *OR* (OU) PT:”COMPARATIVE STUDY” *OR* (OU) “ESTUDO COMPARATIVO” *OR* (OU) “ESTUDIO COMPARATIVO” *OR* (OU) MH:V03.250$ *OR* (OU) PT:”EVALUATION STUDIES” *OR* (OU) “ESTUDOS DE AVALIAÇÃO” *OR* (OU) “ESTUDIOS DE EVALUACION” *OR* (OU) MH:V03.400$ *OR* (OU) PT:”FOLLOW-UP STUDIES” *OR* (OU) SEGUIMENTO$ *OR* (OU) “ESTUDIOS DE SEGUIMENTO” *OR* (OU) MH:E05.318.760.500.750.500.350$ *OR* (OU) MH:N05.715.360.775.175.250.350$ *OR* (OU) MH:N06.850.520.450.500.750.350$ *OR* (OU) MH:”PROSPECTIVE STUDIES” *OR* (OU) “ESTUDOS PROSPECTIVOS” *OR* (OU) “ESTUDIOS PROSPECTIVOS” *OR* (OU) MH:E05.318.760.500.750.500.650$ *OR* (OU) MH:N05.715.360.775.175.250.630$ *OR* (OU) MH:N06.850.520.450.500.750.650$) *OR* (OU) (Tw:clin$ *AND* (Tw:trial$ *OR* (OU) Tw:ensa$ *OR* (OU) Tw:estud$ *OR* (OU) Tw:experim$ *OR* (OU) Tw:investiga$)) *OR* (OU) ((Tw:singl$ *OR* (OU) Tw:simple$ *OR* (OU) Tw:doubl$ *OR* (OU) Tw:doble$ *OR* (OU) Tw:duplo$ *OR* (OU) Tw:trebl$ *OR* (OU) Tw:trip$) *AND* (Tw:blind$ *OR* (OU) Tw:cego$ *OR* (OU) Tw:ciego$ *OR* (OU) Tw:mask$ *OR* (OU) Tw:mascar$)) *OR* (OU) Mh:placebos *OR* (OU) Tw:placebo$ *OR* (OU) (Tw:random$ *OR* (OU) Tw:randon$ *OR* (OU) Tw:casual$ *OR* (OU) Tw:acaso$ *OR* (OU) Tw:azar *OR* (OU) Tw:aleator$) *AND NOT* (E NÃO) (Ct:animal *AND NOT* (E NÃO) (Ct:human and Ct:animal)) #3. #1 *AND* (E) #2 Estratégia de busca para Clinicaltrials.gov *Dupuytren Contracture* (Contratura de Dupuytren) Estratégia de busca para International Clinical Trials Registry Platform (ICTRP) *Dupuytren Contracture* (Contratura de Dupuytren)
